# The Preparation and Characterization of Pyrolysis Bio-Oil-Resorcinol-Aldehyde Resin Cold-Set Adhesives for Wood Construction

**DOI:** 10.3390/polym9060232

**Published:** 2017-06-18

**Authors:** Xueyong Ren, Hongzhen Cai, Hongshuang Du, Jianmin Chang

**Affiliations:** 1 MOE Key Laboratory of Wooden Material Science and Application, Beijing Forestry University, Beijing 100083, China; cjianmin168@126.com; 2 Yunnan Provincial Key Laboratory of Wood Adhesives and Glued Products, Southwest Forestry University, Kunming 650227, China; 3 College of Agricultural Engineering and Food Science, Shandong University of Technology, Zibo 255000, China; chzh666666@126.com; 4 Wood Material Science and Engineering Key Laboratory of Jilin Province, Beihua University, Jilin 132013, China; jldhs@sina.com

**Keywords:** biomass pyrolysis bio-oil, resorcinol-aldehyde resin, cold-set adhesive, wood construction

## Abstract

Resorcinol-formaldehyde (RF) resin is a kind of excellent exterior-grade wood structural adhesive, which can be conveniently cold-set for various applications. In order to decrease the production cost, pyrolysis bio-oil from renewable bioresources was used to replace resorcinol to synthesize the bio-oil-resorcinol-aldehyde (BRF) resin. The effect of replacing resorcinol with bio-oil on the properties, bonding performance, and characterization of resorcinol-aldehyde resin was comparatively investigated. A higher solid content and viscosity, albeit a lower shear strength, was found when the replacement ratio of bio-oil increased. The bonding performance of BRF with 10 and 20 wt % bio-oil was close to that of the pure RF resin. However, the trends of being less cross-linked, more easily decomposed, but more porous were found when the substitution ratio of bio-oil was higher than 20 wt %. Interestingly, it was found that the wood failure values of the BRF resins with bio-oil of no more than 20 wt % were slightly higher than that of the pure RF resin. On the whole, BRF resins with 20 wt % bio-oil is recommended as a wood structural adhesive, comprehensively considering the bio-oil substitution ratio and resin properties. The results obtained here showed that pyrolysis bio-oil is a promising green raw material for the production of RF resin with lower cost.

## 1. Introduction

Wood construction, which has many advantages, such as providing natural, environmental protection and saving energy, has long been considered favorable. Recently, the contemporary construction of tall buildings from wood timber, the only significant building material that is grown, in whole or in part, suggests a growing interest in the potential for building with wood at a scale not previously attainable [1]. Given the rapid development of wood construction, the requirement of cold-set structural adhesive for on-site bonding is growing quickly. Epoxies, polyurethanes, and resorcinol-formaldehyde are common types of adhesive used for on-site bonding, and their characteristics and prospects were previously reviewed by Pizzo et al. [2].

Resorcinol-formaldehyde (RF) resin is one of the mostly used thermosetting adhesives in the production of exterior-grade wood structural materials. Due to the presence of the very reactive resorcinol moiety, RF resin is able to set at ambient temperature. A study on the effects of adhesive types (resorcinol-formaldehyde, RF; polyurethane/aqueous emulsion polymer, PU/AEP; resorcinol–formaldehyde/soy-isolate, RF–Soy), wood species (keruing, southern pine and Douglas-fir), and curing temperatures (ambient, 26 to 35 °C; elevated, 43 to 49 °C) on the strength and durability of a finger-joint was conducted by Vrazel et al. [3]. The results showed that the RF adhesive exhibited the best flexural and tensile strength in the three kinds of adhesive systems evaluated.

Since resorcinol is expensive, produced only in a few locations around the world, and is the key factor in the cost of RF adhesives, phenol and other counterparts are used in RF resins to reduce product costs. Phenol-resorcinol-formaldehyde (PRF) resins of low resorcinol content were prepared by introducing small amounts of urea as a branch, while still maintaining an acceptable pot-life and curing rate at room temperature [4]. Tannins, being phenolic in nature, can be chemically modified and reacted with formaldehyde in the same way formed adhesives harden at ambient temperature in an industrially significant time, and have been used as a raw material to replace resorcinol to prepare the tannin–resorcinol–formaldehyde resin for a long time [5,6,7,8].

Pyrolysis bio-oil, generated by the fast pyrolysis of renewable lignocellulosic biomass, is the cheapest liquid fuel produced from biomass today [9]. Bio-oil, which contains many valuable chemical components, such as phenols, aldehydes, sugar, furan, and acid, has great potential as a feedstock for the fine and bulk chemical products, such as adhesives in particular [10]. An investigation [11] on bio-oils generated from microwave-assisted low-temperature pyrolysis toward aluminum bonding showed bio-oil itself to have adhesive properties, and maximum tensile strengths of bonding between two aluminum plates, from approximately 2520 (bio-oil from spruce wood chips) to 2300 N (bio-oil from waste paper), were observed.

The phenolic-rich bio-oil, generated by a vacuum pyrolysis process, was used to replace part (25 wt % and 35 wt %) of the phenol in a PF resin for oriented strand board (OSB), an exterior grade wood composite product [12]. Bio-oil derived from the pyrolysis of palm kernel shells was also used to synthesize the PF resin, substituting for fossil phenol by up to 25 wt % [13]. Due to the high reactivity and cost efficiency, urea-formaldehyde (UF) resins are widely used in the wood industry, but they tend to emit free and carcinogenic formaldehyde during the application of UF-bonded materials. Wood-derived bio-oil was successfully used to decrease formaldehyde emissions from UF resins during the making of wood-based panels [14]. Bio-oil was pretreated by acetone and then reacted with epoxy for wood bonding, and it was possible to replace the epoxy with bio-oil of as much as 50 wt % while satisfying usage requirements [15,16]. Pyrolysis bio-oil was also used to synthesize the bio-oil phenol formaldehyde (BPF) resins for glass fiber (GF)-reinforced BPF (GF/BPF) resin composites. It was found that high-performance GF/BPF composites could be successfully prepared using the BPF resin with the bio-oil addition of 20 wt % based on the hand lay-up process [17].

Nowadays, the development of environmentally wood adhesives using renewable bioresources is an important goal in the wood industry. Previous studies showed that pyrolysis bio-oil, generated from renewable resources, containing chemical-active compounds, has been successfully used to prepare the interior and thermal-curing adhesives or composites. However, to the best of the author’s knowledge, using bio-oil to synthesize cold-set resin adhesives has rarely been reported. The aim of this study was to investigate the effect of replacing resorcinol with bio-oil on the properties of resorcinol-aldehyde resin, which was expected to be used as a cold-set adhesive for wood construction. A series of bio-oil-resorcinol-aldehyde resins with different substitution ratios of bio-oil were prepared and tested. Chemical structural analysis of both the pure RF resin and bio-oil-resorcinol-aldehyde (BRF) resin were comparatively conducted using a solvent resistance test, FTIR, TGA, and SEM.

## 2. Materials and Methods

### 2.1. Materials

Wood pyrolysis bio-oil was produced at the Institute of Wood-Based Materials (Beijing Forestry University, Beijing, China), which operates a fluidized bed pyrolysis system with a capacity of 1 kg/h of biomass feedstock [18]. Larch wood, collected from Xiao Hinggan Mountains, Heilongjiang Province, China, was used as the feedstock. The water content of bio-oil was 25%, as determined by Karl Fischer titration. The relative chemical composition of bio-oil was 22% phenols, 24% ketone, 20% aldehydes, 16% organic acids, 8% sugars, 4% hydrocarbons, and 6% other compounds, as detected by gas chromatographic-mass spectrometric (GC-MS) analyses on a Shimadzu GC/MS-QP 2010 system (Kyoto, Japan). The resorcinol, sodium hydroxide, methanol, and paraformaldehyde were of reagent grade and used as obtained.

### 2.2. Preparation of BRF Resins

**Figure 1:**
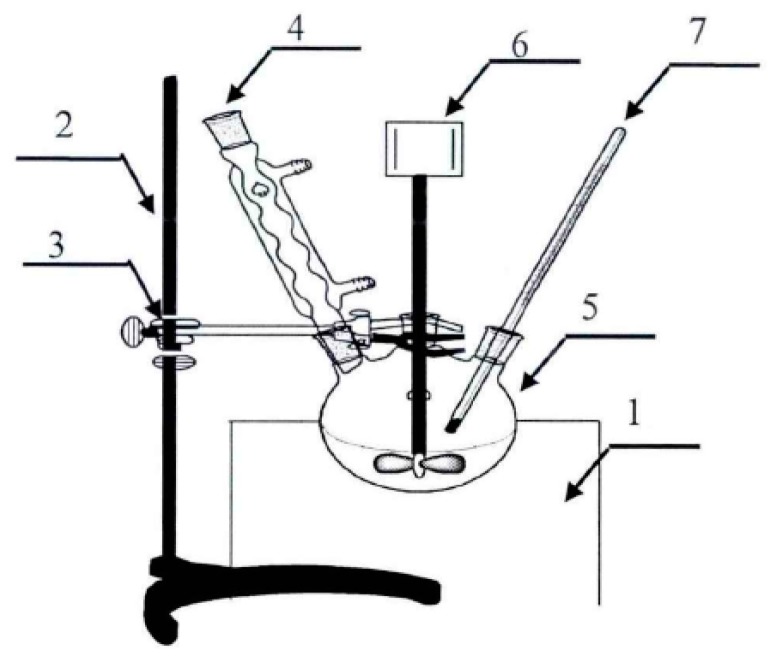
Diagram of three-necked glass reactor apparatus for resin synthesis (1. thermostatic water bath; 2. iron support; 3. tube holder; 4. Allihn condenser; 5. three-necked flask; 6. motor stirrer; 7. thermometer).

In order to study the effect of replacement of resorcinol with bio-oil on the performance of resin adhesive, using a three-necked glass reactor apparatus (Figure 1), BRF resins were prepared by batch copolymerization with bio-oil adding amounts of 0, 10, 20, 30 and 40 wt % (in relation to the mass of resorcinol), denominated as RF, 10%-BRF, 20%-BRF, 30%-BRF, and 40%-BRF, respectively. The molar ratio of resorcinol to formaldehyde is 1:1.2, and the molar ratio of resorcinol to sodium hydroxide is 1:0.2. The specific steps of preparing the BRF resin samples were as follows:

I. At first, the NaOH solution (30 wt %) was added to a three-necked glass reactor and heated to 50 °C. The reactor was equipped with a reflux condenser and a thermometer. Resorcinol with different replacement ratios by bio-oil was dissolved in the NaOH solution under continuous mechanical stirring. After the completely dissolved of resorcinol, the mixture was cooled to 25 °C.

II. Then, half of the formaldehyde solution (37 wt %) and bio-oil solution in methanol were added and mixed for 10 min while the temperature was kept at 30 °C.

III. Next, the remaining half of the formaldehyde solution (37 wt %) was added. The mixture was mixed for 10 min, while the temperature was kept as 25 °C.

IV. Finally, the mixture was heated to 65 °C and maintained for 1 h, then cooled to 25 °C, and the resin pH was re-adjusted to 9.0 and stored.

### 2.3. Preparation of Wood Glulam Test Specimens Bonded by BRF Resins

**Figure 2:**
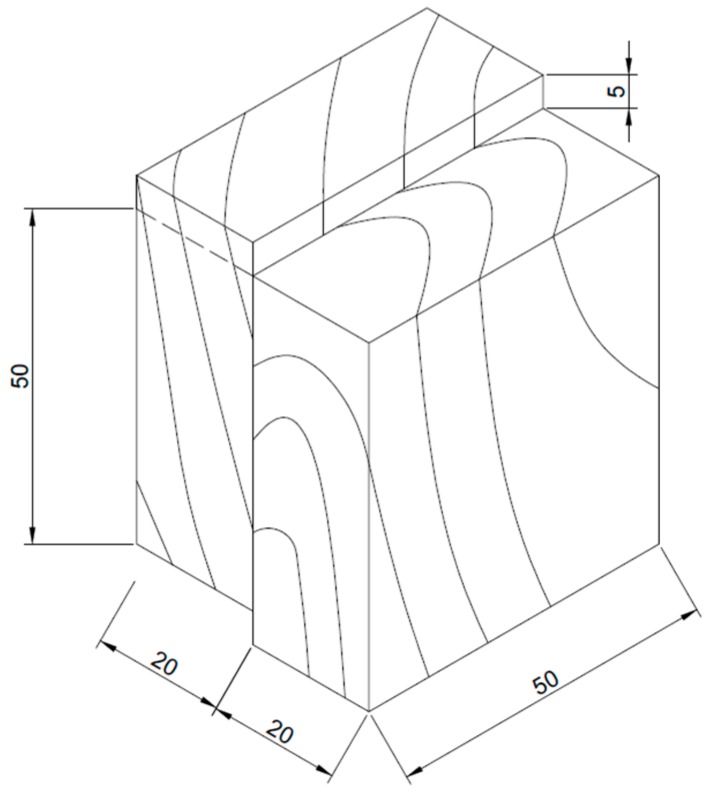
Diagram of wood glulam test specimen for shear strength test.

Wood glulam samples (Figure 2) bonded by RF and BRF resins were prepared to test the bonding performance by the following procedures. Beech wood with a density of 700 ± 50 kg/m^3^ and a water content of 12% was used as the raw material for glulam.

I. At first, the beech wood was cut into blocks following the grain direction with a dimension of 55 mm × 50 mm × 20 mm. The blocks were polished by sand paper and used within 24 h.

II. Then, 15% fine paraformaldehyde powder (200 mesh) as hardener was added into the BRF resin and mixed well to prepare the final adhesives.

III. Next, the final adhesive mixture was spread on the surface of beech wood blocks with a dosage of 200 g/m^2^ and displayed for 10 min.

IV. Finally, two beech wood blocks were assembled together under a pressure of 1.2 MPa for 5 h at ambient temperature. After cold pressing, beech wood glulam samples were preserved for 3 days before the test.

### 2.4. Characterization of BRF Resins

The pH value was measured with a PHS-3B pH meter (Shanghai Precision and Scientific Instrument Co, Ltd, Shanghai, China). The viscosity and solid content of the BRF resins were determined according to the Chinese National Standard, GB/T 14074-2006 (Testing Methods for Wood Adhesives and Their Resins).

The bonding performance of resin was determined in accordance with the Chinese National Standard, GB/T 26899-2011 (Structural Glued Laminated Timber). The dry and wet shear strength, dry and wet wood failure, total delamination rate after cold and boiling water soak were measured. The glulam samples were treated under wet conditions of 24 h cold water soak and 4 h boiled water soak. A universal mechanical testing machine (AG-100KN-MO, Shimadzu Corporation, Kyoto, Japan) was used to perform the shear strength test.

In order to investigate the cross-linking level and structural stability, solvent resistance experiment was conducted on the cured resin using cold water, boiling water, and acetone as the extraction solvent. Water extraction was performed at both conditions of cold distilled water (25 °C) for 24 h and boiled water for 4 h. Acetone extraction was conducted for 6 h (4 solvent cycles per hour) by a Soxhlet Extractor. The weight loss (WL, wt %) was calculated by the weight difference of resin before and after solvent extraction.

The cured resins of BRF and RF were characterized comparatively by ATR-FTIR, TGA, and SEM analysis, so as to understand the effect of replacement of resorcinol with bio-oil on the structure of RF resin. The BRF and RF resins were mixed with paraformaldehyde powder and then cured at the same conditions with wood glulam production process to prepare the cured resins samples. The ATR spectra were recorded in the transmission mode between 4000 and 650 cm^−1^ using a PerkinElmer Frontier FT-IR Spectrometer. Analytical pyrolysis was conducted at a heating rate of 20 °C/min from ambient temperature to 900 °C in a nitrogen atmosphere using a thermo-gravimetric analyzer (Q500, TA instruments, New Castle, DE, USA). TA Instruments Universal Analysis 2000 software (TA instruments, New Castle, DE, USA) was used to analyze the TGA data. Curves of thermogravimetry (TG) and derivative thermogravimetry (DTG) were plotted and analyzed. Scanning Electron Microscope (SEM) was used to obtain SEM images of cured BRF and RF resins by a Hitachi SU8010 instrument (Hitachi Limited, Tokyo, Japan).

## 3. Results and Discussion

### 3.1. Properties of the BRF Resins

Similar to the brown color of the RF resin, the BRF resins was also brown with slightly darker than pure RF resin due to the introduction of bio-oil. Table 1 shows the pH value, solid content, and viscosity of the RF and BRF resins.

The pH value of the BRF resin with a replacement of bio-oil of up to 20 wt % was very close to that of the pure RF resin. However, when more than 20 wt % bio-oil added into the RF resin, the pH value of resin decreased due to the acidity of bio-oil. The acidic compounds in bio-oil are mainly organic acid, which are hard to be neutralized quickly.

All the solid content of the resins obtained here was more than 50 wt %, and a high solid content was beneficial for the initial adhesion properties of adhesive during the gumming and bonding process. The BRF resins exhibited a higher solid content than pure RF resin, and its solid content increased as more bio-oil was used. This phenomenon may be due to two reasons: one reason is the high-molecule staff in bio-oil, namely pyrolytic lignin (mainly the oligomers from lignin) [19], and another reason is the highly complex compounds of bio-oil, such as phenols, aldehydes, and long-chain ketones, which mainly participate in the condensation reactions to quickly form large complicated molecules or long branches during the synthesis process of RF resin.

For most of the types of resin, viscosity and solid content were close to each other. Therefore, the solid content of the BRF resins also increased as the bio-oil replacement ratio increased. It is noteworthy that the viscosities of 10%-BRF and 20%-BRF are similar to the RF resin, while the viscosities of 30%-BRF and 40%-BRF are markedly larger than theirs.

### 3.2. Bonding Performance of the BRF Resins

In order to characterize the bonding performance of the BRF resin, the shear strength, wood failure, and total delamination rate of glulam at dry and wet states (24 h cold water soak and 4 h boiled water soak) were measured and are shown in Table 2.

Pure RF resin shows excellent bonding performance regarding the good mechanical properties and water-resistant ability. The bonding properties of 10%-BRF was quite close to that of the RF resin, while the 20%-BRF resin showed a slight declined properties regarding the shear strength and total delamination rate. When bio-oil of more than 20 wt % was added to the BRF resin, its bonding performance began to markedly decrease. It was expected that active compounds in bio-oil, such as phenols and aldehydes, would participate in the synthetic reactions of RF resin. However, the content of these active compounds in bio-oil is limited, as there is a certain concentration of water and high-molecule oligomers. When a proper amount of bio-oil, 20 wt % here in this study, was used to replace resorcinol, a good BRF resin was obtained without losing too much bonding strength and water resistance. After that, the shear strength, wood failure, and total delamination rate of the BRF resins began to drop sharply, as the inert and less active compounds hindered the synthetic reactions between resorcinol and formaldehyde, or, when more bio-oil was used as reactant, caused resorcinol to drop to insufficient amounts.

It is interesting that the wood failure values of 10%-BRF and 20%-BRF were slightly higher than that of the pure RF resin. Wood failure, defined as the area percentage of wood damage on the overall test specimen surfaces, is an important index for evaluating the bonding performance of adhesive and glulam. Higher wood failure indicated that the 20%-BRF resin demonstrated superior bonding compared with the pure RF resin. Due to the introduction of pyrolysis bio-oil, containing many water-soluble compounds, the BRF resin was more hydrophilic than the pure RF resin. In addition, the BRF resins have a higher solid content than does the RF resin, as shown in Table 1, which is optimal for the formation of glue nail in porous materials. Wood material has a porous and hydrophilic surface. The BRF resin exhibited more hydrogen bonding, a higher solid content, and a superior glue nail effect than did the pure RF, more strongly adhering to wood surface.

The Chinese National Standard of GB/T 26899-2011 requires that the shear strength be more than 6.0 MPa and that the total delamination rate be less than 5%. Based on this standard, the BRF resin with no more than 30% replacement of bio-oil satisfied the performance requirement. The shear strength and total delamination rate of the 40%-BRF resin under wet conditions was below the standard. On the whole, comprehensively considering the bio-oil replacement ratio and bonding performance, the 20%-BRF resin is recommended for wood structural adhesives. Enriching or separating the active compounds in bio-oil, such as phenols and aldehydes, is needed to prepare a good bio-oil-based RF resin, if a higher proportion of bio-oil is used in resin synthesis. In addition, the formulation and synthetic process parameters also need to be optimized for the production of the BRF resin, with a high ratio of bio-oil used.

### 3.3. Solvent Resistance

The solubility of a polymer, determined by solvent resistance experiment, not only provides an indication of the cross-linking density in cured resins, but can also be used to evaluate the environmental tolerance of the cured resins and can offer some valuable data for its industrial application. The weight loss of BRF after cold water, boiling water, and acetone extraction tests are shown in Table 3.

The WL of the RF resin under conditions of cold water, boiled water, and acetone extraction were 6.89, 9.52 and 16.58 wt %, respectively. Acetone extracted the highest amount of non-structural materials from the cured resin due to its great solvency to organic resins. The WL of the RF resin increased as more bio-oil was added to the resin. It should be noted that, when the replacement ratio of bio-oil was no more than 20 wt %, the WL of the BRF resin was still low and quite close to that of the pure RF resin. However, after a substitution with bio-oil of more than 20 wt %, the WL of the BRF resin increased considerably. This is because of the increased concentrations of hydrophilic and polar compounds in the unreacted bio-oil, such as carboxylic acids and alcohols, which tend to be absorbed to the solvent and being separated out [20]. Unsaturated aldehyde ketone conjugate materials with some aromatic rings, and conjugated polyene, were reported in extracts from a bio-oil/epoxy resin after solvent extraction, demonstrated by a UV–Vis study [15].

### 3.4. FTIR Spectrum Analysis

**Figure 3:**
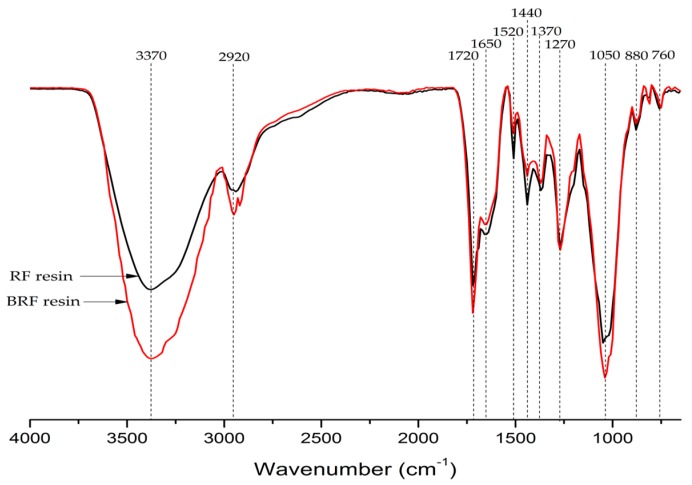
FTIR spectrum of 20%-BRF and RF resins.

To investigate and compare the chemical structure of BRF and RF resins, the 4000–650 cm^−1^ infrared spectrum is shown in Figure 3. The 20%-BRF resin had an FTIR spectrum similar to that of the RF resin, indicating that the BRF resin exhibited a molecular structure similar to that of the RF resin.

Based on previous studies [15,17,21,22], the main peaks in the IR spectrum of the RF and BRF resins were identified and discussed as following. The presence of a large intense bond at 3370 cm^−1^ corresponds to O–H stretching vibrations, which is a promising indication of the water/acid/alcohol. The peaks near 2920/1720 correspond to the aliphatic C–H and C=O stretching, respectively. Both peaks of 1650 and 1520 cm^−1^ showed an aromatic C=C stretching vibration, while the deformations of aliphatic CH_2_ and CH_3_ were assigned as the peaks of 1440 and 1370 cm^−1^, respectively. The existence of aliphatic CH_2_ indicated the long-chain structure in both RF and BRF resins. The peak at 1270 cm^−1^ was related to the aromatic CO– and phenolic –OH stretching. The peak at 1050 cm^−1^ is well known as the stretching of alcohol C–O or aliphatic ether C–O. The aliphatic CH deformation was indicated by the peaks of 880 and 760 cm^−1^.

The main differences between the BRF and RF resins were located at the peaks around 3370, 2920, 1720, 1520, 1440, and 1050 cm^−1^. In comparison with the spectrum of the RF resin, the higher intensity of bands at 3370, 2920, 1720, and 1050 cm^−1^ for the BRF resin markedly increased, possibly indicating the existence of some unreacted substances in the BRF resin, such as alcohol, aliphatic hydrocarbon, and carbonyl compounds. These possible functional groups and compounds suggested here were proposed based on the comprehensive analysis of several IR peaks, the existed compounds in bio-oil, as well as the chemical reaction mechanism of resorcinol-aldehyde resin. Due to the replacement of resorcinol with bio-oil, the intensity of bands attributed to the methylene bridged bond (1440 cm^−1^) and the aromatic C=C (1650 and 1520 cm^−1^) for the BRF resin became slightly weaker than that of the RF resin, showing that less cross-linking of the main chain structure was obtained in the BRF resin. This finding can explain the reason for the lower bonding strength of the BRF resin compared to that of the pure RF resin.

### 3.5. TGA Measurement

**Figure 4:**
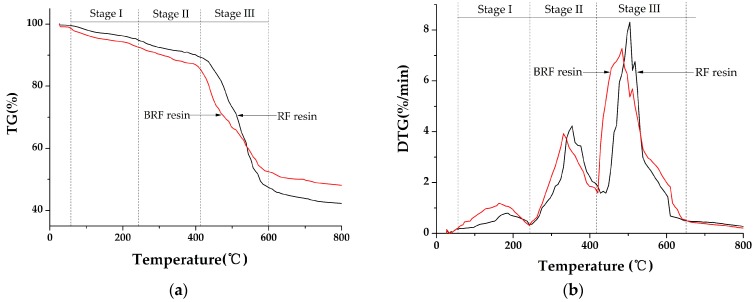
Thermogravimetry (TG) (**a**) and derivative thermogravimetry (DTG) (**b**) curves of 20%-BRF and RF resins.

The thermogravimetry (TG) and derivative thermogravimetry (DTG) curves of the 20%-BRF and RF resins are shown in Figure 4. The DTG curves for both of the resins similarly displayed three thermal decomposition events, which reflected a three-step degradation of the RF resin similar to that proposed for the bio-oil PF resin: post-curing, thermal reforming, and ring stripping [23].

In this regard, the first maximum thermal event at 160–180 °C could be ascribed to the post-curing reactions of the RF and BRF resins. The post-curing process involved the removal of water and terminal groups, as well as condensation reactions or further crosslinking. The second event, the thermal reforming to break the bridged methylene linkage between the resin units, occurred in the temperature range from 240 to 400 °C; the third event, in the range 400–650 °C, was the breakdown of the ring network in the main structure.

The carbon residue content of the BRF resin (48 wt %) was higher than that of the RF resin (42 wt %), which may be explained by the higher solid content of bio-oil. It was reported that a carbonaceous and macro-porous solid material was formed during the degradation process of bio-oil [24], which could hinder the decomposition rate of the BRF resin. As a result, the highest weight-loss rate (7.3 wt %/min) of the BRF resin was lower than of the RF resin (8.3 wt %/min). In addition, it was found that the three stages of BRF degradation process shifted to a slightly lower temperature than that of the BR resin, which means that the structural stability of the BRF resin may be mildly weaker than the RF resin.

### 3.6. SEM Analysis

**Figure 5:**
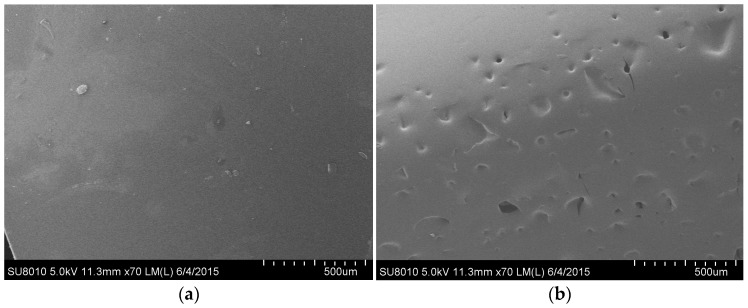
The scanning electron microscope (SEM) photograph of the (**a**) RF and (**b**) 20%-BRF resins.

The SEM images of the 20%-BRF and RF resins is shown in Figure 5. In Figure 5a, the surfaces of cured RF resin appeared to be smooth and flat, indicating its uniform, dense, and compact resin microstructure, as the high reactivity of resorcinol with formaldehyde in the synthesis process. After the primary synthesis reaction, A-stage RF resin was obtained, which has a linear structure that is water- or alcohol-soluble. Under the curing effect of a hardener or heat, the A-stage resin changed into B-stage and eventually C-Stage, which has a three-dimensional cubic structure that is insoluble and un-meltable.

The BRF resin showed a flat microstructure similar to that of the pure RF resin, but with small pores on the surface (Figure 5b). These small pores may have been caused by the evaporating and separating out of the unreacted substances with a low boiling-point, which were contained in the BRF resin but did not exist in the main steady structure of the resin. During the curing process, the further cross-linking of the molecular chain squeezed out the weak and unstable compounds or groups in the resin, also subjected to the side effect of the heat from the curing exothermic process, which eventually slotted the resin surface by the sputtering of the micro-molecules produced.

This slightly porous surface of the BRF resin could be optimal for bonding with wood, a porous and non-uniform material. It is believed that BRF resins tend to form more glued nails into the wood surface than pure RF resins, which is confirmed by the wood failure data in Table 2. However, it needs to be noted that this effect is limited and may be detrimental to the structural stability of the resin in the bonding interface when there are too many pores produced in the BRF resin as more bio-oil is introduced into the resin.

## 4. Conclusions

Pyrolysis bio-oil was used to replace resorcinol to synthesize the cold-set resin as a wood structural adhesive. The effect of replacing resorcinol with bio-oil on the bonding properties and the characterization of resorcinol-aldehyde resin was comparatively investigated. As the replacement ratio of bio-oil to resorcinol increased, an RF resin with a higher sold content and viscosity, but a lower shear strength, was obtained. Interestingly, it was found that the wood failure values of BRF with bio-oil of no more than 20 wt % were slightly higher than those of the pure RF resin. The structural characterization by FTIR, TGA, and SEM showed that, with the introduction of bio-oil, the RF resin was not as cross-linked, more easily decomposed, but more porous. The bonding properties of the bio-oil RF resin with 20 wt % bio-oil is close to that of the pure RF resin, which, comprehensively considering the bio-oil ratio, bonding performance, and solvent resistance, is recommended for wood structural adhesives.

## Figures and Tables

**Table 1:** Properties of resorcinol-formaldehyde (RF) and bio-oil-resorcinol-aldehyde (BRF) resins.

Resin	pH	Solid Content (wt %)	Viscosity (mPa·s)
RF	9.00 ± 0.15	53.5 ± 0.4	560 ± 10
10%-BRF	9.00 ± 0.18	54.3 ± 0.8	570 ± 11
20%-BRF	8.93 ± 0.12	54.9 ± 0.5	575 ± 15
30%-BRF	8.76 ± 0.09	57.7 ± 1.1	610 ± 18
40%-BRF	8.58 ± 0.24	59.8 ± 0.6	638 ± 14

**Table 2:** Bonding performances of the RF and BRF resins.

Resin	Shear Strength(MPa)			Wood Failure			Total Delamination Rate	
	Dry	24 h Cold	4 h Boil	Dry	24 h Cold	4 h Boil	24 h Cold	4 h Boil
RF	9.65 ± 0.25	7.88 ± 0.19	7.69 ± 0.18	90 ± 2	75 ± 2	70 ± 3	2.03 ± 0.03	2.35 ± 0.04
10%-BRF	9.36 ± 0.18	7.65 ± 0.16	7.75 ± 0.17	92 ± 3	76 ± 3	69 ± 4	1.98 ± 0.05	2.55 ± 0.08
20%-BRF	8.95 ± 0.14	7.32 ± 0.14	7.33 ± 0.15	95 ± 4	79 ± 4	75 ± 3	2.25 ± 0.13	1.98 ± 0.12
30%-BRF	7.82 ± 0.21	6.56 ± 0.20	6.38 ± 0.19	85 ± 4	68 ± 5	63 ± 2	4.25 ± 0.12	3.85 ± 0.09
40%-BRF	7.53 ± 0.12	5.90 ± 0.15	5.85 ± 0.22	75 ± 5	62 ± 3	52 ± 3	5.68 ± 0.35	5.35 ± 0.28

**Table 3:** Weight loss (WL) of cured resins extracted by cold water, boiled water, and acetone.

Resin	Weight Loss (wt %)		
	Cold Water Extraction	Boiled Water Extraction	Acetone Extraction
RF	6.89 ± 0.21	9.52 ± 0.19	16.58 ± 0.35
10%-BRF	7.25 ± 0.15	9.95 ± 0.26	17.12 ± 0.52
20%-BRF	7.64 ± 0.26	10.25 ± 0.34	18.67 ± 0.42
30%-BRF	9.59 ± 0.45	13.56 ± 0.24	19.35 ± 0.68
40%-BRF	12.56 ± 0.33	18.45 ± 0.42	24.88 ± 0.71

## References

[1] Ramage H.M., Burridge H., Busse-Wicher M., Fereday G., Reynolds T., Shah U.D., Wu G.L., Yu L., Fleming P., Densley-Tingley D. (2017). The wood from the trees: The use of timber in construction. Renew. Sustain. Energy Rev..

[2] Pizzo B., Smedley D. (2015). Adhesives for on-site bonding: Characteristics, testing and prospects. Constr. Build. Mater..

[3] Vrazel M., Sellers T. (2004). The effects of species, adhesive type, and cure temperature on the strength and durability of a structural finger-joint. For. Prod. J..

[4] Pizzi A., Scopelitis E. (1993). The chemistry and development of branched PRF wood adhesives of low resorcinol content. J. Appl. Polym. Sci..

[5] Pizzi A., Roux D.G. (1978). The chemistry and development of Tannin-based weather- and boil-proof cold-setting and fast-setting adhesives for wood. J. Appl. Polym. Sci..

[6] Kreibich E.R., Hemingway W.R. (1985). Condensed tannin-resorcinol adducts in laminating adhesives. For. Prod. J..

[7] Grigsby W., Warnes J. (2004). Potential of tannin extracts as resorcinol replacements in cold cure thermoset adhesives. Holz Roh-und Werkst..

[8] Zhou X., Pizzi A. (2013). Tannin-resorcinol-aldehyde cold-set wood adhesives with only formaldehyde as harder. Eur. J. Wood Prod..

[9] Vispute T.P., Zhang H.Y., Sanna A., Xiao R., Huber G.W. (2010). Renewable chemical commodity feedstocks from integrated catalytic processing of pyrolysis oils. Science.

[10] Hosseinnezhad S., Fini H. E., Sharma K. B., Basti M., Kunwa B. (2015). Physiochemical characterization of synthetic bio-oils produced from bio-mass: A sustainable source for construction bio-adhesives. RSC Adv..

[11] Zhang Z.R., Macquarrie J.D., Clark H.J., Matharu S.A. (2015). Green materials: Adhesive properties of bio-oil derived from various biorenewable waste streams: From wood to paper to paper deinking residue. ACS Sustain. Chem. Eng..

[12] Chan F., Riedl B., Wang X.M., Lu X., Amen-Chen C., Roy C. (2002). Performance of pyrolysis oil-based wood adhesives in OSB. For. Prod. J..

[13] Choi G.G., Oh S.J., Lee S.J., Kim J.S. (2015). Production of bio-based phenolic resin and activated carbon from bio-oil and biochar derived from fast pyrolysis of palm kernel shells. Bioresour. Technol..

[14] Li B., Zhang J.Z., Ren X.Y., Chang J.M., Gou J.S. (2014). Preparation and characterization of bio-oil modified urea-formaldehyde wood adhesives. Bioresources.

[15] Liu Y., Via K.B., Pan Y.F., Cheng Q.Z., Guo H.W., Auad L.M., Taylor S. (2017). Preparation and characterization of epoxy resin cross-linked with high wood pyrolysis bio-oil substitution by acetone pretreatment. Polymers.

[16] Celikbag Y., Robinson J.T., Via K.B., Adhilari S., Auad L.M. (2015). Pyrolysis oil substituted expoxy resin: Improved ratio optimization and crosslinking efficiency. J. Appl. Polym. Sci..

[17] Cui Y., Chang J.M., Wang W.L. (2016). Fabrication of glass reinforced composites based on bio-oil phenol formaldehyde resin. Materials.

[18] Ren X.Y., Gou J.S., Wang W.L., Li Q., Chang J.M., Li B. (2013). Optimization of bark fast pyrolysis for the production of phenol-rich bio-oil. Bioresources.

[19] Bai T.T., Chang J.M., Ren X.Y., Wang W.L., Liu Q.Y., Zhang Z.T. (2015). TG-FTIR analysis of pyrolytic lignin extracted from different kinds of bio-oil. J. Biobased Mater. Bioenergy.

[20] Gagnon M., Roy C., Riedl B. (2004). Adhesives made from isocyanate and pyrolysis oils for wood composites. Holzforschung.

[21] Hosseinnezhad S., Fini H.E., Sharma K.B., Basti M., Kunwar B. (2015). Physiochemical characterization of synthetic bio-oils produced from bio-mass: A sustainable source for construction bio-adhesives. RSC Adv..

[22] Sasakia C., Wanakaa M., Takagib H., Tamurac S., Asadaa C., Nakamura Y. (2013). Evaluation of epoxy resins synthesized from steam-exploded bamboo lignin. Ind. Crops Prod..

[23] Cheng S.N., Yuan Z.S., Anderson M., Leitch M., Xu C.B. (2012). Synthesis of biobased phenolic resins/adhesives with methylolated wood-derived bio-oil. J. Appl. Polym. Sci..

[24] Ren X.Y., Meng J.J., Moore M.A., Chang J.M., Gou J.S., Park S.K. (2014). Thermogravimetric investigation on the degradation properties and combustion performance of bio-oils. Bioresour. Technol..

